# Do costs of reproduction differ between the sexes of dioecious plants?

**DOI:** 10.1093/aob/mcaf178

**Published:** 2025-08-08

**Authors:** Marcel E Dorken

**Affiliations:** Department of Biology, Trent University, 1600 West Bank Drive, Peterborough, ON, Canada K9L 0G2

**Keywords:** Life-history trade-offs, somatic costs of reproduction, demographic costs of reproduction, cost function, sex allocation

## Abstract

**Background:**

Costs of reproduction arise from fitness-based trade-offs between current and future reproduction. Because the average fitness of females and males is constrained to be equal, and because costs of reproduction are paid in units of fitness, the extent to which reproductive costs can differ between the sexes has been called into question.

**Scope:**

Using expressions that incorporate the trade-off between current and future reproductive fitness, I examine whether costs of reproduction can in principle differ between the sexes. These expressions clarify the types of evidence that could be used to infer divergence in costs of reproduction between the sexes.

**Key Results:**

Costs of reproduction can differ between the sexes only insofar as females and males can diverge in their expected future reproductive potential. If the two sexes differ in their future reproductive potential and one sex prioritizes future opportunities over current output, that sex should experience lower costs of reproduction.

**Conclusions:**

The expected future reproductive potential of plants is driven by their schedules of survival and reproduction, which are difficult to study for long-lived plants. However, it may be possible to infer differences in the costs of reproduction between females and males by combining two pieces of evidence: sex differences in (1) the magnitude of trade-offs between reproduction and growth or survival; and (2) the propensity to produce offspring after previous bouts of reproduction (e.g. via differences in post-reproductive growth or survival). Evidence for (1) and (2) exists widely, but they have rarely been studied together in dioecious populations, leaving little solid evidence regarding how often costs of reproduction differ between females and males.

## INTRODUCTION

Contrasting opinions have recently emerged regarding the extent to which reproductive costs differ between females and males in dioecious plant populations. On the one hand, the average fitness of females and males in a dioecious population is constrained to be equal (Fisher, 1930), which, as argued by Midgley (2022), should constrain reproductive allocations and their associated costs to also be equal. On the other hand, Pannell (2025) contends that reproductive allocations and costs are not interchangeable concepts, and thus the constraint of equal fitness between the sexes does not necessarily lead to equal costs of reproduction. Indeed, Pannell (2025) highlights many examples of divergent reproductive effort and sexual dimorphism in life history that might reflect sex-specific costs. However, despite these observations, whether reproductive costs truly differ between the sexes in dioecious plants is not as clear-cut as often implied in previous reviews of sexual dimorphisms and reproductive costs. While the possibility for sex-specific reproductive costs has been established for hermaphrodites (Crowley, 2008), theoretical exploration for dioecious species remains limited. Early work suggested that reproductive costs should be equal in males and females (Williams, 1975, p. 120), a view supported by Midgley (2022). Moreover, empirical support for sex-specific differences in reproductive costs is often less robust than frequently claimed (Dorken *et al*., 2025). In this review, I aim to clarify whether reproductive costs can differ between the sexes in dioecious plants and to identify the types of evidence that could be used to support these differences.

The proposal that costs of reproduction might differ between the sexes of dioecious plants has a long history, rooted in early observations such as Darwin’s note on sex-dimorphic strawberries: ‘much more vital force is expended in the production of ovules and fruit than in the production of pollen’ (Darwin, 1877, p. 293). Subsequent reviews of sexual dimorphisms (Lloyd and Webb, 1977; Delph, 1999; Barrett and Hough, 2013) and of the costs of reproduction (Obeso, 2002) have summarized studies – typically of single reproductive episodes – that report greater reproductive effort for females than for males, in some cases alongside measures of performance (e.g. measures of photosynthesis, growth and survival) that differed between the sexes. However, a recurring challenge in this body of work, highlighted even in early reviews, has been to demonstrate that such observed differences translate into divergent costs of reproduction between the sexes. As Lloyd and Webb (1977, p. 206) emphasized, ‘The mere fact that only females spend considerable resources on seed and fruit maturation as well as on achieving fertilization does not require a greater reproductive effort by females.’ The failure to clearly distinguish between measures of reproductive allocations and reproductive costs has contributed to lingering conceptual difficulties.

Beyond the challenge of distinguishing allocations from costs, another conceptual obstacle for determining whether the sexes can differ in their reproductive costs is how measures of plant performance – such as growth or photosynthetic rates – can reflect differences in fitness between females and males. On the one hand, using metrics of performance to serve as proxy measures of fitness is practical, particularly for the study of dioecious plants most of which are long-lived (Sakai and Weller, 1999), making more direct measurements of fitness difficult to obtain. On the other hand, the sexes do not directly compete against each other for fitness in the same way that they compete against individuals of the same sex (e.g. during mating under sexual selection). Because costs of reproduction arise from a trade-off between current and future reproductive fitness (Williams, 1966; Obeso, 2002; Dorken *et al*., 2025), such morphological or physiological differences between females and males reflect divergent costs of reproduction if they also reflect differences in fitness between the sexes. As pointed out by Midgley (2022), how this could occur is not at first glance clear. For example, if plant size is used as a proxy measure of fitness and males are on average larger than females, the argument that larger size for males is evidence for lower costs of reproduction also implies that males have greater average fitness than females – a conclusion that would seem to fly in the face of the requirement of equal average fitness of females and males. This conceptual challenge, together with limited empirical support for sex differences in the magnitude of reproductive trade-offs (Dorken *et al*., 2025), means that whether the sexes can diverge in their costs of reproduction remains a largely open question.

## COSTS OF REPRODUCTION

Costs of reproduction arise from trade-offs between current versus future reproduction (Williams, 1966). If resources allocated to present reproduction diminish an organism’s growth or survival, this can consequently reduce future reproductive output. Costs of reproduction are therefore intrinsically linked to an organism’s life history (i.e. its schedule of reproduction and survival), and thus its lifetime fitness. Indeed, reproductive costs, as originally conceived, are defined by their effects on fitness, specifically as the reduction to an individual’s future reproductive success (Williams, 1966; Bell, 1980). While many studies, particularly in plants, adopt a broader, more practical interpretation – viewing any trade-off involving reproduction as a ‘cost’ due to limited resources (e.g. nutrients, photosynthates; Obeso, 2002, Dorken *et al*., 2025) – this still implicitly relies on the assumption that such trade-offs affect fitness.

Most formal definitions of fitness adopt Fisher’s (1930) definition: the expected contribution of an individual’s genes to future generations. That is, fitness is determined by the transmission of genes via the production of offspring. Alternative definitions that incorporate gene transmission through time (as opposed to generations, e.g. via survival and clonal propagation; Zhang and Wang, 1994; Pedersen and Tuomi, 1995; Zhang, 2006) do not fit as well with the concept of costs of reproduction. If growth (including clonal growth) or survival contribute directly to current fitness, the distinction between current and future reproduction dissolves, precluding any sensible consideration of reproductive costs. This point was raised in the two papers related to this Perspective: Midgley (2022) argued that because fitness shapes all aspects of plant life histories, whether directly or indirectly, all resources are ultimately allocated to reproduction. However, as pointed out by Pannell (2025), lumping other components of the life history such as growth or survival into reproduction amounts to casting aside key concepts in life-history theory: understanding trade-offs among growth, survival and reproduction is central to various topics in ecology and evolution (Salguero-Gómez *et al*., 2016; Züst and Agrawal, 2017; Hutchings, 2021), including, costs of reproduction (Dorken *et al*., 2025). Maintaining the distinction between life-history components and defining fitness as transgenerational gene transmission through offspring clearly positions growth and survival as indirect contributors to fitness via their effects on future reproductive opportunities, not as direct contributors to current reproduction. Indeed, this understanding is inherent to the many studies of reproductive costs that have investigated trade-offs between reproduction and survival or growth (including clonal growth; Dorken *et al*., 2025). Studying trade-offs between growth (or survival) and reproduction implies that growth is an alternative to current reproduction, and therefore that it contributes indirectly to fitness.

**Fig. 1.**
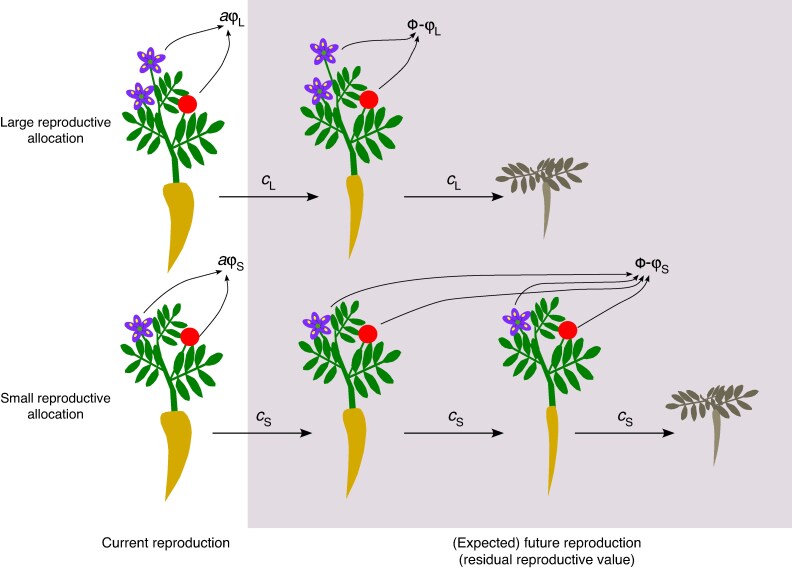
Costs of reproduction arise from the trade-off between current and future reproduction. This trade-off filters through plant life histories, with allocations to reproduction reducing the amount of resources available for growth and survival (Dorken *et al*., 2025). Plants with a larger allocation to reproduction have higher fitness in the current round of reproduction but experience stronger trade-offs with growth and survival, indicated here as a reduction in the size of a perennating organ. Plants with a smaller allocation to reproduction have lower fitness in the current round of reproduction ($a\phi_{S}<a\phi_{L}$) but live longer and, as depicted here, can expect to survive for an additional round of reproduction. In this simplified diagram, each flower (purple) and fruit (red circle) contributes equally to lifetime fitness (*Φ*) and to reproductive costs (*c*). As a result, higher fitness from current reproduction for plants with larger reproductive allocations comes at a cost of lower expected future reproductive fitness (lower residual reproductive values) compared to plants with smaller reproductive allocations. For simplicity, this diagram depicts hermaphrodite plants.

Using fitness as the metric for estimating costs of reproduction, a plant that increases its allocation to reproduction in one flowering season increases its fitness via (current) reproduction at the cost of opportunities to gain fitness in the future. This future reproductive fitness is known as its ‘residual reproductive value’ – its reproductive value after accounting for previous investments in reproduction, where reproductive value is the expected reproductive success of an individual of a given age and sex (Fisher, 1930; Pianka and Parker, 1975). This change in residual reproductive value arising from allocations current to reproduction is the foundation for the original definition of the costs of reproduction (Williams, 1966). Following William’s (1966) notation, a trade-off between fitness gained from a current bout of reproduction *ϕ* and total lifetime reproductive fitness *Φ* yields a residual reproductive value of R=Φ−ϕ (Fig. 1). Females make an allocation to reproduction that increases current reproductive fitness by a factor *a* at a cost to future reproduction that reduces their residual reproductive value by a factor *c*:


$$\Phi_{F}=a_{F}\phi_{F}-c_{F}R_{F}.$$

Similarly, the effect of current reproduction on male fitness is:


$$\Phi_{M}=a_{M}\phi_{M}-c_{M}R_{M}.$$

At evolutionary equilibrium, the value of *Φ* is maximized with selection against individuals that deviate from this value (Williams, 1966). Moreover, because every offspring has exactly one mother and one father, the total genetic contribution to the next generation from all females in a population must equal the total genetic contribution from all males (Fisher, 1930). So, while individual reproductive success can vary greatly within each sex (e.g. one male fertilizing multiple females), the average fitness (i.e. the average genetic contribution to the next generation) of males and females across the entire population must be equal. Accordingly, we can set $\Phi_{F}=\Phi_{M}$ and solve for $c_{F}$:


$$c_{F}=\frac{c_{M}R_{M}-a_{M}\phi_{M}+a_{F}\phi_{F}}{R_{F}}.$$

This expression helps clarify the conditions under which costs of reproduction may differ between the sexes. Recalling that *ϕ* is a component of *R*, costs of reproduction differ between females and males if the average contribution to the next generation of plants in each round of reproduction (aϕ) differs between females and males from the same cohort (i.e. groups of plants of similar age in a population of perennial plants with overlapping age classes). In other words, costs of reproduction can only differ between the sexes if females and males differ in terms of the fitness gained from current versus future reproduction.

**Fig. 2.**
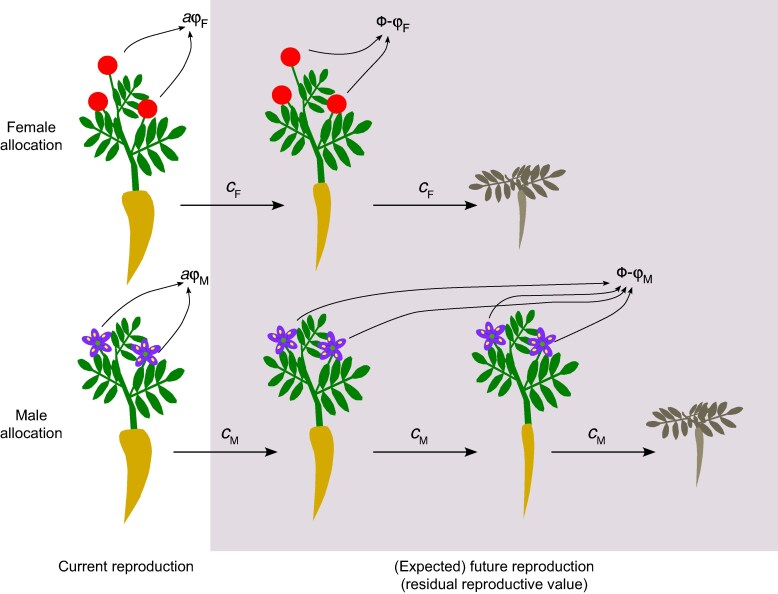
Costs of reproduction may differ between the sexes of dioecious plants if they differ in their residual reproductive values ($\Phi_{F}-\phi_{F}\neq \Phi_{M}-\phi_{M}$). Males produce flowers (purple) and females produce flowers (not depicted) and fruits (red circles). Here, males are assumed to have lower costs of reproduction and higher residual reproductive values than females. As for Fig. 1, each fruit and flower represents equal contributions to fitness and to reproductive costs (*c*). Accordingly, as depicted, lifetime fitness for males is the same as for females ($\Phi_{F}=\Phi_{M}$), but males can still have lower costs of reproduction than females by having greater residual reproductive values.

This analysis also clarifies the scope for different reproductive costs between the sexes to be expressed across plant life histories. For polycarpic (iteroparous) plants, which undergo multiple reproductive bouts, there are ample opportunities for the expression of reproductive costs, and therefore for sex-specific divergence in these costs. For these plants, divergence in reproductive costs between females and males implies that, at least for a given cohort of plants, the fitness gained from current reproduction for females differs from that of males ($a_{F}\phi_{F}\neq a_{M}\phi_{M}$). Under these conditions, residual reproductive values for females ($R_{F}$) do not need to equal those of males ($R_{M}$) even if the total expected lifetime reproductive fitness of females and males are equal (Fig. 2). Concluding that costs of reproduction differ between females and males follows from identifying differences in fitness contributions from a single round of reproduction of females and males, which further implies differences in the residual reproductive values of females and males.

Estimating the fitness of females and males during one or more reproductive bouts is exceedingly difficult and rarely conducted (but see Horvitz *et al*., 2010; Miller *et al*., 2012). As a result, directly testing for differences between the sexes in reproductive fitness, let alone differences in their future reproductive fitness, is unlikely to be practical, particularly for long-lived plants. However, the frequently documented sexual dimorphism in life history across dioecious plants can provide insights into differences in the costs of reproduction between the sexes (Lloyd and Webb, 1977; Delph, 1999; Barrett and Hough, 2013). For example, if males have lower costs of reproduction than females, this requires them to also have greater residual reproductive values than females. In this scenario, males would have forgone opportunities to gain fitness in one round of reproduction, which can only be favoured by natural selection if they also have greater expected fitness gains in the future. Observations that males have higher post-reproductive survival or growth would be consistent with divergence in the reproductive values of females and males after a bout of reproduction.

In contrast to polycarpic plants, a straightforward application of the model to monocarpic (semelparous) plants implies that average costs of reproduction for females and males must be balanced. For such plants, which have a single, terminal reproductive event, there are no opportunities for additional reproduction in the future, so $\Phi_{F}=a_{F}\phi_{F}$ and $\Phi_{M}=a_{M}\phi_{M}$. Because of the equality of fitness for the two sexes ($\Phi_{F}=\Phi_{M}$), there is no scope for divergence in the values of aϕ or *R* between the sexes (because Φ=aϕ and $R_{F}=R_{M}=0$), and therefore no scope for divergence in costs of reproduction between the sexes ($c_{F}=c_{M}$), at least under the assumptions made in the model.

While the model’s implication of equal reproductive costs between the sexes for monocarpic plants is sensible, it rests on the assumption that reproductive allocations have immediate effects on future reproductive fitness. In natural populations, this assumption is too simplistic, and relaxing this assumption opens the possibility for the expression of sex-specific reproductive costs for plants with a single reproductive event. In particular, there is a temporal gap between the initial allocation of resources to reproduction and the final maturation and dispersal of offspring. So, even for monocarpic plants, resource investment into early reproductive structures might reduce the success of the current reproductive bout by affecting the chance that a plant survives until all seeds are fully mature. Research on annual *Arabidopsis thaliana* exemplifies this, demonstrating that reproductive costs can arise even in plants with rapid life cycles. For *A. thaliana*, plants with moderate investments into herbivore defence have greater reproductive success than poorly defended plants in spite of an underlying trade-off between reproduction and defence (Mauricio and Rausher 1997). The temporal gap between the initial allocation of resources to reproduction and the dispersal of offspring therefore opens the possibility for the expression of costs of reproduction in dioecious plants with rapid life cycles, including the expression of sex-specific differences in reproductive costs (Harris and Pannell, 2008; Sánchez Vilas and Pannell, 2011).

## HOW DIVERGENT COSTS OF REPRODUCTION BETWEEN THE SEXES ARE EXPRESSED

That differences in the costs of reproduction between the sexes are reflected by differences in residual reproductive values is not a new insight. For example, male burying beetles (*Nicrophorus vespilloides*) have greater residual reproductive values than females and, accordingly, are less likely than females to incur reproductive costs in a given bout of reproduction (Ward *et al*., 2009). For these beetles, males were more likely to abandon small broods than females, a tactic associated with greater reproductive success for males in subsequent rounds of reproduction. Similar divergences in residual reproductive values, and therefore in reproductive costs, also seem possible for dioecious plants. For example, as already noted, males are frequently larger and grow faster than females, particularly in woody plants (Obeso, 2002; Barrett and Hough, 2013). If fertility scales with plant size and presumably therefore the amount of resources available for reproduction, as generally expected (particularly for dioecious plants; Charnov *et al*., 1976; Seger and Eckhart, 1996; Charlesworth, 1999), then faster growth for males might lead to greater residual reproductive values for males than for females. If so, this would be consistent with lower reproductive costs for males than for females, particularly among younger cohorts of plants (Charlesworth and Leon, 1976).

Differences in patterns of growth and reproductive effort that are consistent with divergence in residual reproductive values between females and males have been reported for at least two plants. First, for the long-lived herbaceous plant *Borderea pyrenaica* females have higher reproductive effort than males, particularly among younger cohorts of plants and are the only sex to express detectable trade-offs between current and future reproduction (assessed using a flower-removal treatment; Sherman *et al*., 2019). Males, by contrast, maintain lower reproductive effort than females, at least until they reach older ages and never express a detectable trade-off between current and future reproduction. Both of these points are consistent with greater residual reproductive values for males compared to females, and therefore lower costs of reproduction. Second, for the shorter-lived herbaceous plant *Silene latifolia*, males have been inferred to have greater reproductive costs than females (Delph *et al*., 2005). So, here one would expect females to have higher residual reproductive values than males. Indeed, for *Silene latifolia*, evidence for greater costs of reproduction in males compared to females coincides with evidence that after reproduction males are smaller than females (Delph and Meagher, 1995) and with data consistent with the possibility that males have lower survival than females (Carroll and Mulcahy, 1993; Taylor, 1994; Delph *et al*., 2005). Smaller size and lower survival should yield lower residual reproductive values for males compared to females, supporting the inference of lower costs of reproduction for females compared to males (Delph *et al*., 2005).

Sexual dimorphism in populations of dioecious plants is well documented (Lloyd and Webb, 1977; Delph, 1999; Obeso, 2002; Barrett and Hough, 2013), but does not on its own indicate that costs of reproduction differ between the sexes. In particular, sexual dimorphisms in life history and in reproductive trade-offs are necessary but not sufficient evidence that the costs of reproduction differ between females and males. For example, females and males in dioecious populations of the clonal plant *Sagittaria latifolia* are sexually dimorphic in terms of clonal expansion, with females expanding slower than males because of stronger trade-offs between reproduction and clonal growth (Van Drunen and Dorken, 2012). However, despite apparent differences in life history-related traits that appear to be driven by reproductive trade-offs, shoot-level sex ratios are on average balanced at 50:50 (Yakimowski and Barrett, 2014) and there are no apparent differences between the sexes in plant growth or patterns of survival when transplanted into a natural wetland (Dorken and Barrett, 2003). Similar growth and survival for females versus males are inconsistent with divergent residual reproductive values for females versus males for *Sagittaria latifolia*. This example provides a note of caution: sexually dimorphic life histories, even when combined with divergent reproductive trade-offs – at least by some measures – are not on their own sufficient evidence that costs of reproduction differ between the sexes.

Compelling evidence for sex-specific differences in the costs of reproduction emerges when females and males are divergent both in the magnitude of reproductive trade-offs and in their post-reproductive schedules of reproduction and survival. In particular, only sex differences in traits that affect future opportunities for reproduction are implicated as being potential markers of divergent costs of reproduction between the sexes. As implied when describing the *B. pyrenaica* and *Silene latifolia* examples above, the conclusion that females and males differ in their costs of reproduction because of divergent life histories was supported by evidence for differences in reproductive trade-offs between the sexes, and in differences in the likelihood of future reproduction via divergent schedules of reproductive effort for *B. pyrenaica* and divergent patterns of survival for *Silene latifolia*. To date, studies that link divergent life histories and reproductive trade-offs are relatively rare (Dorken *et al*., 2025), leaving little in the way of solid evidence that costs of reproduction can and do frequently differ between the sexes of dioecious plants.

## CONCLUSION

Costs of reproduction arise from fitness-based trade-offs between current and future reproduction. Accordingly, differences in the costs of reproduction between females and males may arise whenever there is scope for divergent opportunities for future reproductive fitness between the sexes. Evidence of such divergence requires documenting not only that reproductive trade-offs differ between the sexes, but also that females and males are dimorphic for traits associated with schedules of reproduction and/or survival in a manner that can be expected to affect their future reproductive success. These two lines of evidence have rarely been obtained together for dioecious plants and, as a result, there are few clear examples of differences in the costs of reproduction between females and males. Although it may be possible for costs of reproduction to differ between the sexes, it remains unclear how often this difference occurs.

## AI assistance

AI tools were used to refine earlier drafts of the manuscript to improve the clarity of certain sentences. No original text was generated using AI.
